# The impact of crystal size and temperature on the adsorption-induced flexibility of the Zr-based metal–organic framework DUT-98

**DOI:** 10.3762/bjnano.10.169

**Published:** 2019-08-20

**Authors:** Simon Krause, Volodymyr Bon, Hongchu Du, Rafal E Dunin-Borkowski, Ulrich Stoeck, Irena Senkovska, Stefan Kaskel

**Affiliations:** 1 Chair of Inorganic Chemistry, Technische Universität Dresden, Bergstrasse 66, 01062 Dresden, Germany; 2 Centre for Systems Chemistry, Stratingh Institute for Chemistry, University of Groningen, Nijenborgh 4, 9747 AG Groningen, The Netherlands; 3 Ernst Ruska-Centre for Microscopy and Spectroscopy with Electrons, Forschungszentrum Juelich GmbH, 52425 Juelich, Germany; 4 Central Facility for Electron Microscopy (GFE), RWTH Aachen University, 52074 Aachen, Germany

**Keywords:** crystal engineering, crystal size, flexible metal–organic frameworks, MOFs, water adsorption

## Abstract

In this contribution we analyze the influence of adsorption cycling, crystal size, and temperature on the switching behavior of the flexible Zr-based metal–organic framework DUT-98. We observe a shift in the gate-opening pressure upon cycling of adsorption experiments for micrometer-sized crystals and assign this to a fragmentation of the crystals. In a series of samples, the average crystal size of DUT-98 crystals was varied from 120 µm to 50 nm and the obtained solids were characterized by X-ray diffraction, infrared spectroscopy, as well as scanning and transmission electron microscopy. We analyzed the adsorption behavior by nitrogen and water adsorption at 77 K and 298 K, respectively, and show that adsorption-induced flexibility is only observed for micrometer-sized crystals. Nanometer-sized crystals were found to exhibit reversible type I adsorption behavior upon adsorption of nitrogen and exhibit a crystal-size-dependent steep water uptake of up to 20 mmol g^−1^ at 0.5 *p*/*p*_0_ with potential for water harvesting and heat pump applications. We furthermore investigate the temperature-induced structural transition by in situ powder X-ray diffraction. At temperatures beyond 110 °C, the open-pore state of the nanometer-sized DUT-98 crystals is found to irreversibly transform to a closed-pore state. The connection of crystal fragmentation upon adsorption cycling and the crystal size dependence of the adsorption-induced flexibility is an important finding for evaluation of these materials in future adsorption-based applications. This work thus extends the limited amount of studies on crystal size effects in flexible MOFs and hopefully motivates further investigations in this field.

## Introduction

In the past 20 years, research in the area of metal–organic frameworks (MOFs) has delivered various record-holding materials in terms of surface area [1] and gas storage [2] and has also given rise to unprecedented adsorption phenomena [3] often associated with structural transitions. An increasing number of the so-called flexible MOFs are being reported and their use in the areas of storage [4], separation [5] and sensing [6] of gases is being evaluated; their structural flexibility and adsorption behavior can be manipulated by applying chemical functionalization to the ligand [7] and metal cluster [8]. However, recent examples progressively illustrate the manipulation of the adsorption properties of switching adsorbents by variation of size [9–13] and morphology [14] of the crystals, without changing the composition of the MOF. The reports on the MOF MIL-53 indicate an impact of crystal morphology and size on the structural contraction upon solvent removal [15–16]. In a recent report, we demonstrated the suppression of adsorption-induced contraction in the MOF DUT-49 (where the name DUT is derived from Dresden University of Technology) upon downsizing of the crystals below 1 µm [9] and loss of flexibility upon downsizing of DUT-8(Ni) crystals [17–18]. In a similar fashion, the suppression of gate opening in ZIF-8 upon crystal downsizing was previously reported [10–13] and Kitagawa and co-workers reported on a shape–memory effect of the gate-opening transition upon crystal downsizing in a pillared-layer MOF [19]. Although these reports demonstrate the influence of crystal size on the structural behavior of flexible MOFs, the materials for which this phenomenon was reported differ in terms of topology, the nature of the structural transitions and the resulting adsorption behavior. In fact, it appears to be possible that some reported nonflexible MOFs can exhibit flexibility if their crystals are synthesized at a specific size or with a specific morphology. It is thus of crucial importance to extend investigations on the crystal size effects in flexible and nonflexible MOFs to further define these phenomena and potentially postulate a global mechanism.

## Results and Discussion

**Figure 1:**
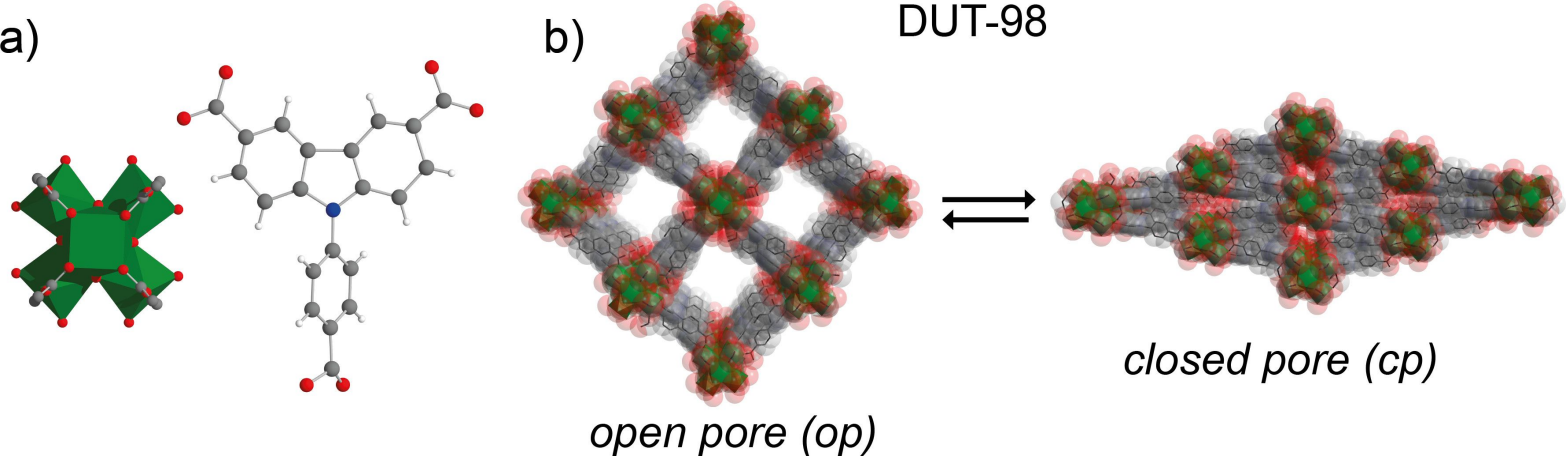
a) Zr-oxo cluster (green) and organic ligand CPCDC (9-(4-carboxyphenyl)-9*H*-carbazole-3,6-dicarboxylate) applied in the construction of DUT-98; b) crystal structure transformation of DUT-98 open pore (*op*) to closed pore (*cp*) configuration.

In this contribution we report on the impact of crystal size on adsorption- and temperature-induced structural transitions of DUT-98, a flexible Zr-based MOF. The structure of DUT-98 is comprised of 1D supramolecular building blocks (SBBs), which consist of Zr_6_ clusters interconnected by 3,6-carbazole dicarboxylate ligands [20]. An additional benzoate functionality of the ligand connects the SBB into a 3D framework with a structure (Figure 1) similar to MIL-53 [21–22].

**Figure 2:**
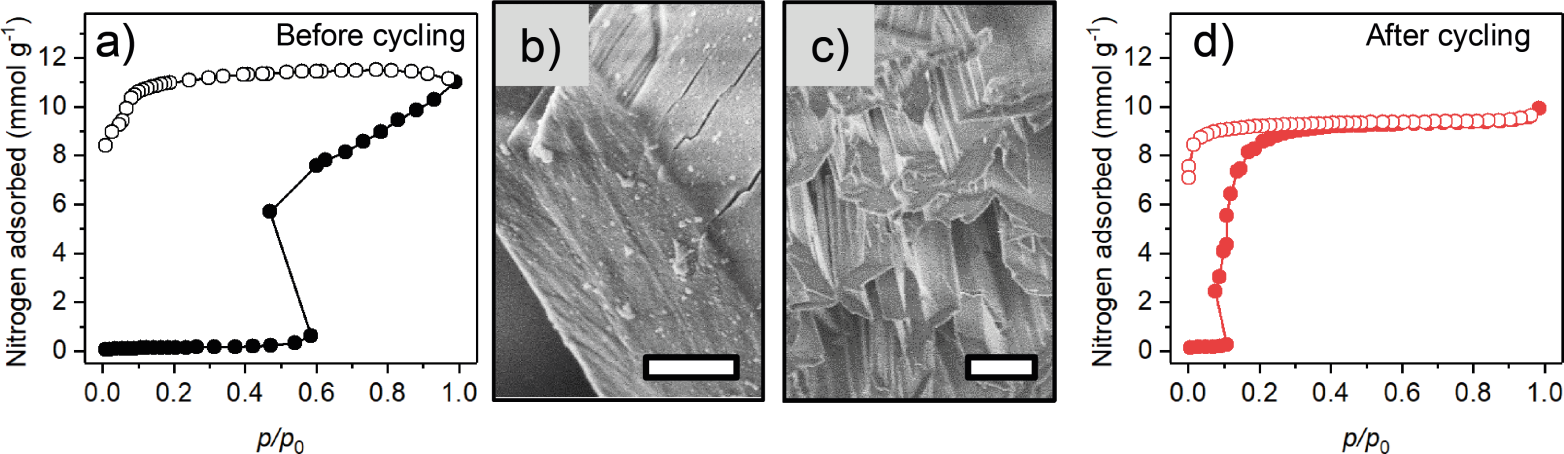
Nitrogen adsorption isotherms at 77 K (a,d) and SEM images (scale bars 1 µm) (b,c) of DUT-98(1) before (a,b) and after (c,d) adsorption experiments with nitrogen, CO_2_, *n*-butane, and vapors of alcohols and hydrocarbons (for details on the adsorption experiments see [23]). Closed symbols: adsorption, open symbols: desorption.

Upon removal of the solvent molecules from the pore channels by supercritical activation, the wine-rack-type channels of DUT-98 are contracted almost completely, reducing the gas-accessible pore volume by 98%. By adsorption of various guest molecules, including N_2_ (at 77 K), CO_2_ (at 195 K), *n*-butane (at 273 K), and various alcohols (at 298 K), the pores of the DUT-98*cp* (*cp* = closed pore) MOF reopen at a defined gate-opening pressure (*p*_go_), giving rise to an isotherm with distinct steps that are typical for gate-opening MOFs. Upon cyclic adsorption/desorption, the structural transition and the structure of the corresponding *op* and *cp* phase remains (Supporting Information File 1, Figure S3); however, a shift of *p*_go_ towards a lower pressure is observed, indicating that the material properties change upon adsorption and desorption of various gases and vapors (Figure 2).

Only few reports on the cycling behavior of flexible MOFs can be found in the literature. Shi et al. reported on a shift of the gate pressure towards higher pressures upon cycling of the nitrogen adsorption/desorption at 77 K in Zn_2_(tdc)_2_(pvq) (H_2_tdc = 2,5-thiophenedicarboxylic acid, pvq = 5-(2-(pyridin-4-yl)vinyl)quinoline) [24]. Similar results are reported by Su et al. on a redox-active flexible MOF [25]. Nitrogen isotherms at 77 K of both materials show a pronounced pressure reduction upon structural opening referred to by the authors as “negative gas pressure”. Such artificial pressure reduction upon structural expansion is also observed in DUT-98 and a few other examples in the literature [23,25–27] and is a clear indication of a metastable adsorption state, as previously described by Kitagawa and co-workers [28].

In a previous work, we addressed changes in the adsorption behavior in flexible MOFs upon cycled *n*-butane adsorption experiments on a series of flexible MOFs with predominantly one-dimensional channels [29]. In that investigation, the *p*_go_ in DUT-8(Ni) and SNU-9 isotherms was found to be shifted to higher pressures upon cycling. In contrast, the cycling of MIL-53 and ELM-11 was found to only slightly impact the adsorption behavior. The analysis of the SNU-9 and DUT-8(Ni) crystals before and after cycling showed deformation and fragmentation of the relatively large crystals compared to the microcrystals of MIL-53 and ELM-11, which were not affected by the cycling. This indicates that larger crystals are more prone to undergo deformation and fragmentation upon repeated contraction/expansion due to the higher number of unit cells merged in a single crystal that have to collectively switch upon structural transition. However, in all reported cases, *p*_go_ was shifted to higher pressures upon cycling in contrast to DUT-98, for which the gate pressure is found to be shifted to lower pressures. The SEM images of the DUT-98 crystals before and after cycling (Supporting Information File 1, Figures S1,S2, Figure 2) show that the rod-shaped crystals of DUT-98*cp* are fragmented into smaller needle-like pieces. Structurally, this indicates a fragmentation along the pore channels, which run along the rods and crystallographic *b*-axis in DUT-98*cp*. Thus, bond cleavage upon fragmentation is expected to occur at the edges of the SBB in DUT-98, which runs in parallel to the pore channels. This fragmentation is likely caused by the stress upon structural reopening during adsorption and contraction during desorption in which the unit cell volume changes by over 140%. The change in the adsorption isotherm as well as the crystal morphology and fragmentation indicate that the adsorption behavior depends on the size of the crystal domains. The question is whether this behavior can also be observed if the crystals were initially synthesized smaller in size?

**Figure 3:**
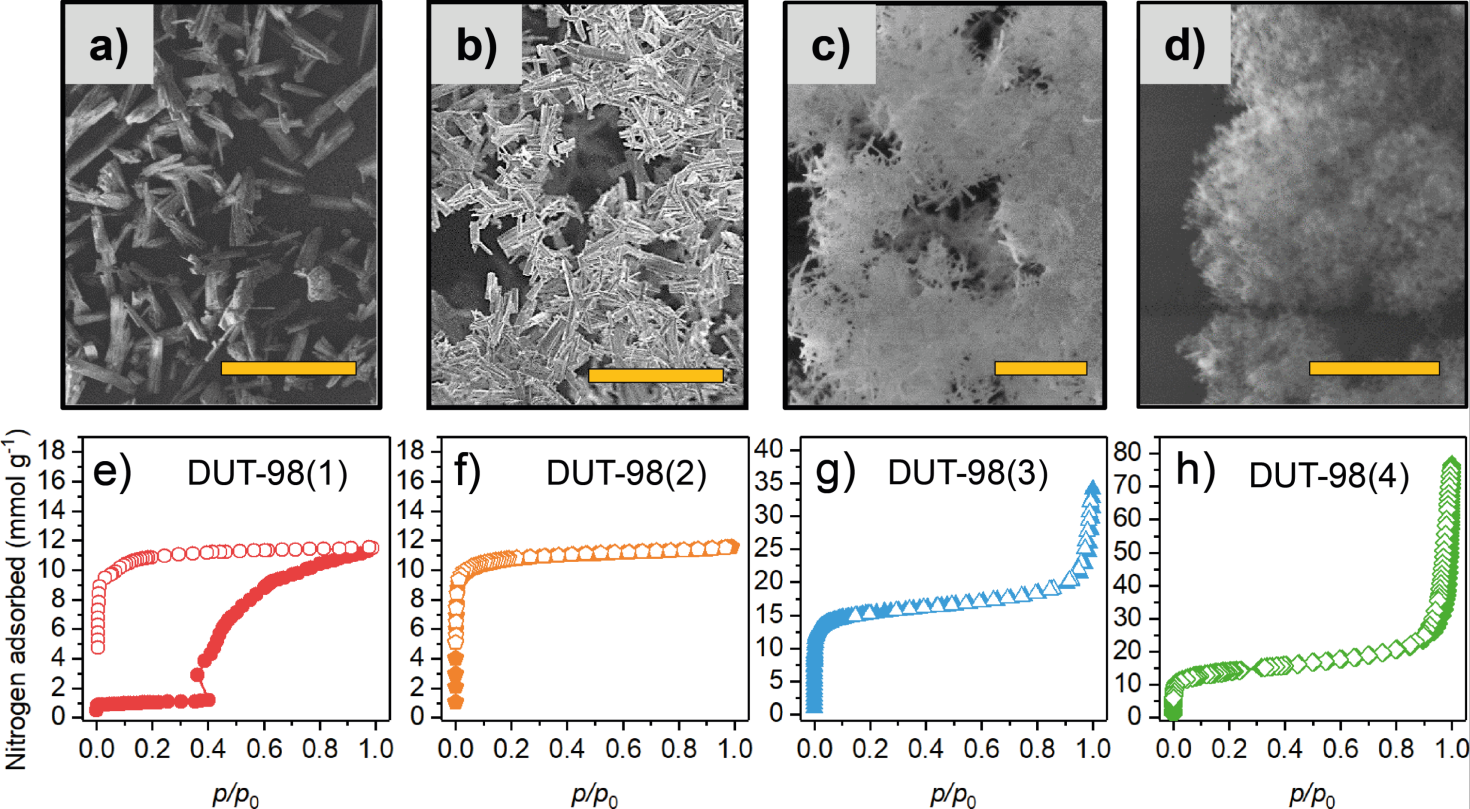
a–d) SEM images and e–h) nitrogen adsorption–desorption isotherms at 77 K for samples DUT-98(1) (a,e), DUT-98(2) (b,f), DUT-98(3) (c,g), and DUT-98(4) (d,h). Scale bars: a) 200 µm, b) 50 µm, c) 1 µm, and d) 500 nm. Closed symbols: adsorption, open symbols: desorption.

Recently it has been shown that the crystal size of Zr-based MOFs can easily be controlled by adding acidic modulators [30–31] or water [32–33] to the reaction mixture in the solvothermal synthesis, which increase or decreases the crystal size, respectively. By changing the solvent, reaction time, concentration of acetic acid, and water content of the reaction mixture, four DUT-98 samples containing particles of different size, namely DUT-98(1)–(4) could be obtained (Figure 3).

**Figure 4:**
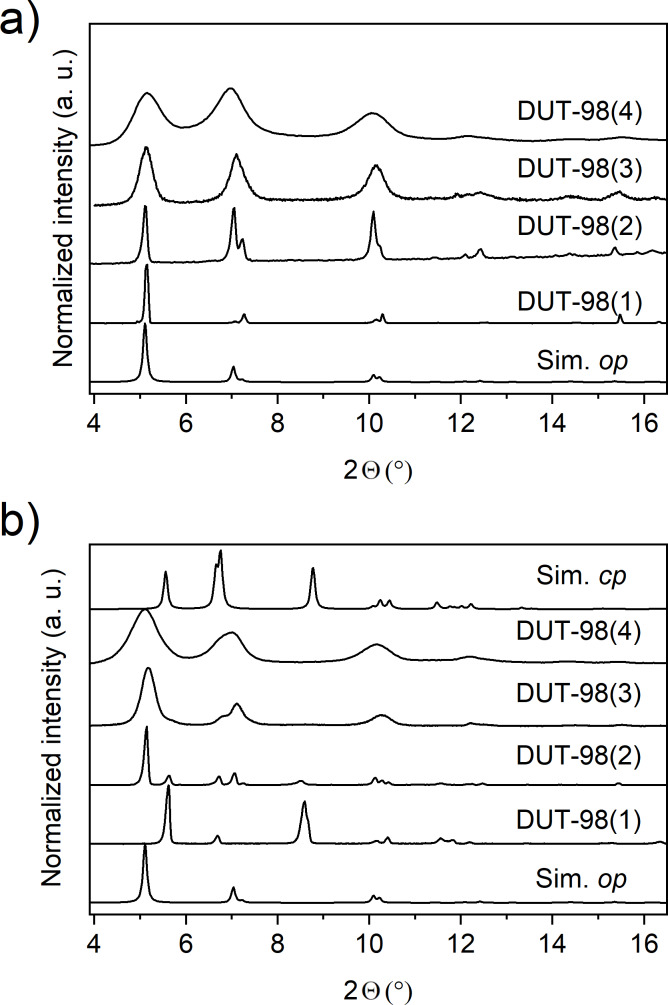
Powder XRD patterns of DUT-98 with varying crystal size a) as-synthesized and b) activated by supercritical solvent removal.

All solvated samples were analyzed by powder X-ray diffraction (PXRD), illustrating the phase purity of the desired DUT-98*op* (open pore) phase (Figure 4). A significant peak broadening is observed for samples 3 and 4, which is indicative of the presence of nanometer-sized crystals. Quantitative analysis of the mean crystal size by applying the Debye–Scherrer method was unsuccessful due to peak overlap and coexistence of multiple phases as described later. The samples were further activated according to the procedure previously reported for DUT-98(1) [20]. Solvent-free white powders, and in the case of DUT-98(4), low-density monolithic structures (similar to aerogels recently reported for gel-like Zr MOFs [34–35]), were obtained. The SEM analysis shows a rod-like crystal morphology with crystal length in the range of 120 µm for DUT-98(1), 10 µm for DUT-98(2), 500 nm for DUT-98(3), and 50 nm for DUT-98(4) (Supporting Information File 1, Figures S4–S7). Thermogravimetric analysis (TGA) of activated DUT-98 samples shows a decrease in the decomposition temperature from 440–420 °C with decreasing crystal size (Supporting Information File 1, Figure S11). However, the general shape of the TGA curves is very similar, indicating the phase purity of the powders. PXRD analysis of the supercritically activated samples shows again peak broadening upon downsizing. However, for DUT-98(1)–(3) samples, a shift in the reflection positions and appearance of new peaks, different from that of the *op* phase, are observed (Figure 4), which was previously assigned to the contraction of DUT-98*op* into DUT-98*cp*.

Interestingly, only DUT-98(1) exhibits complete contraction of the whole bulk, while the other three samples only exhibit peaks of low intensity of the DUT-98*cp* phase and primarily remaining peaks of the *op* phase. Consequently, in the samples with smaller crystals, the majority of crystals or crystal domains remain in the *op* phase and only part of the sample undergoes contraction upon solvent removal by supercritical activation. A quantitative phase analysis of the *op*–*cp* mixture by Rietveld refinement was not possible due to the anisotropy of the crystals and the large peak broadening, leading to an overlap of the reflections of the two phases. To further analyze the nature of the phase mixture, we performed high-resolution transmission electron microscopy (HRTEM) analysis on DUT-98(4). HRTEM was previously applied for the microscopic analysis of defects in Zr-MOFs [36]. Unfortunately, the nanocrystals of DUT-98(3) decomposed during the measurement. By reproducing the synthesis using Hf instead of Zr we obtained DUT-98(Hf) with a crystal length of 130 nm, which demonstrated the same structure as shown by PXRD (Supporting Information File 1, Figure S9). Interestingly, DUT-98(Hf) showed enhanced stability towards the electron beam allowing for detailed microscopic analysis of the nanocrystals and their structure. HRTEM analysis shows uniform pore channels along the rod-shaped nanocrystals with a spacing of the Hf cluster of 1.5 nm, which is in good agreement with the lattice parameter and the inter-cluster distance of the DUT-98*op* crystal structure (Supporting Information File 1, Figure S8). The regular arrangement of the clusters indicates a high symmetry that matches the tetragonal symmetry in DUT-98*op*. In contrast, the DUT-98*cp* phase would exhibit a different microscopic structure due to the lower, monoclinic symmetry. In addition, no missing cluster or linker defects could be detected that are well-known for Zr-MOFs [36–37], demonstrating that the observed behavior does not depend on increasing concentration of lattice defects. Thus, all investigated DUT-98(Hf) crystals exhibited a structure expected for the open phase, which supports the results obtained from PXRD and nitrogen adsorption at 77 K in which the *op* phase is the dominant phase. We thus assume that the presence of the *cp* phase is based on a physical mixture of crystals exhibiting either *op* or *cp* phase and that these phases do not co-exist as domains within a single crystal. This supports the assumption that structural contraction is a cooperative phenomenon that propagates through the whole crystal. To further analyze the porosity and adsorption-induced flexibility, N_2_ adsorption–desorption isotherms at 77 K were recorded on DUT-98(2)–(4) and compared to the initial isotherm of DUT-98(1) (Figure 3e–h).

Interestingly, only DUT-98(1) exhibits a flexible behavior evident by the stepped isotherm and wide hysteresis. DUT-98(2) exhibits type I isotherm, reflecting the microporosity of the MOF. DUT-98(3) and (4) show type I behavior at lower relative pressures and type IV behavior at higher relative pressures, reflecting the microporous nature of the MOF and additional interparticle mesoporosity, respectively. This assumption is supported by comparing the specific micropore volume and specific BET surface area (Supporting Information File 1, Table S2) of DUT-98(2)–(4), which exhibit comparable values with DUT-98(3), showing the highest values of 0.53 cm^3^ g^−1^ and 1303 m^2^ g^−1^, respectively. Interestingly, neither of the materials reach the pore volume value calculated theoretically from the crystal structure of DUT-98*op,* indicating that the *cp* fraction observed in the PXRD pattern does not reopen upon adsorption of nitrogen at 77 K. In this regard, DUT-98(Hf) is found to exhibit similar adsorption properties (Supporting Information File 1, Figure S10) compared to DUT-98(3), which further supports the observations made by PXRD and HRTEM. In fact, neither of the isotherms of DUT-98(Hf) nor DUT-98(2)–(4) show any indication of adsorption-induced flexible behavior, evident by steps or hysteresis in the isotherm. Thus, nitrogen, known to be a rather weakly interacting adsorbate, cannot initiate a structural contraction in downsized crystals of DUT-98.

**Figure 5:**
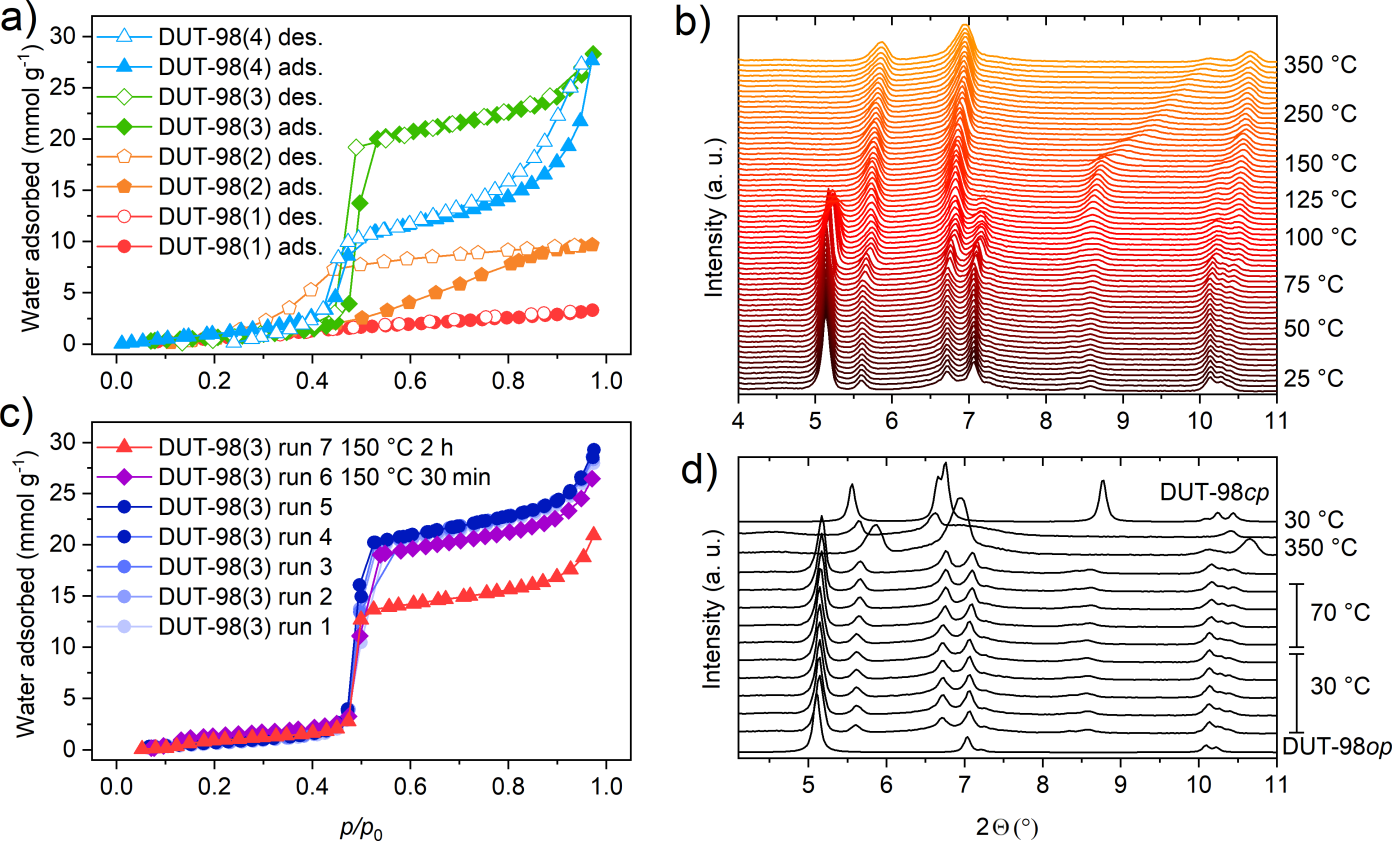
Water physisorption experiments at 298 K on: a) DUT-98(1)–(4) and c) DUT-98(3) after cycling and after thermal treatment. (Filled symbols: adsorption, open symbols: desorption). In situ temperature-variable PXRD of: b) DUT-98(2) and d) DUt-98(2) cycled at lower temperature.

The contraction mechanism in DUT-98(1) was previously shown to depend on pore shrinkage along a reorganization of water molecules within the structure close to the Zr cluster [23]. The diffuse reflectance Fourier transform (DRIFT) spectroscopy results show typical vibrations corresponding to the linker and OH groups of the MOFs in all materials (Supporting Information File 1, Figure S12). To analyze whether adsorption of water can promote contraction in small crystals of DUT-98*op*, water adsorption experiments were conducted at 298 K for DUT-98(2)–(4) (Figure 5a).

In DUT-98(1), no significant adsorption of water can be observed, indicating that structural opening cannot be induced via water adsorption. On the other hand, in DUT-98(2), an increase in uptake is observed around a relative pressure of 0.5, indicating increased adsorption ability. In contrast to other Zr-based MOFs [38], this is a rather high relative pressure for water adsorption, indicating a hydrophobic character of the pore inner surface. In DUT-98(3), a steep increase in water adsorption is observed again at around a relative pressure of 0.5, however the uptake is found to be almost three times higher compared to DUT-98(2). DUT-98(4) exhibits the same steep increase but a wide hysteresis at higher pressure, indicating the dominant contribution of interparticle mesoporosity over the microporosity of the pore channels. Repeated water adsorption experiments on DUT-98(3) show near identical isotherms, supporting the absence of structural transitions and cycling stability under these conditions. The high cycling stability and steep uptake at a relative pressure around 0.5 might make this material an interesting candidate for water capture applications [39–41].

In the original report on DUT-98, an irreversible contraction of the pores upon thermal activation at 80 °C in vacuum was described. An unknown structural transition, different from the contraction to DUT-98*cp*, takes place, and the formation of a high-temperature (DUT-98*ht)* phase was proposed. Although the structure of this phase could not be refined from PXRD and single crystal diffraction data, the obtained PXRD patterns were found to be very similar to the *cp* phase. In addition, DRIFT analysis indicates that the transition is supported by the loss of lattice water molecules [23]. Because the crystal downsizing allows for the preservation of the desolvated DUT-98*op* phase, we investigated the impact of elevated temperature on the structural transition in DUT-98*op* via in situ variable-temperature PXRD. The experiments were conducted under vacuum on supercritically activated DUT-98(2) in the temperature range of 30–350 °C. These conditions are often used for the thermal activation of MOFs and were previously applied in the analysis of DUT-98(1) [23]. In the range of 30–75 °C, no change in the PXRD patterns could be observed and the material could be heated and cooled down within this rage without any indication of structural changes (Figure 5). However, upon further heating, a clear increase in the peak intensity assigned to reflections of DUT-98*ht* is observed. At around 100 °C, the peaks of the *op* phase disappear and a phase-pure *ht*-phase is obtained. At temperatures beyond 150 °C, the peaks at 8.5 and 11.5° exhibit a gradual shift towards a higher diffraction angle, indicating a reduction of the lateral intercluster distance. This would support the proposed enhanced contraction upon heating that is potentially supported by the loss of lattice water not evident in the DRIFT spectra after heating (Supporting Information File 1, Figure S13). Such a *ht*-phase has also been observed for MIL-53, which corresponds to the loss of water from the structure. Attempts to index the obtained patterns failed potentially due to a change in symmetry and the high crystal anisotropy. Nevertheless, the variable-temperature PXRD analysis clearly shows that elevated temperatures initiate a temperature-irreversible structural transition in DUT-98(2), which was also previously observed for DUT-98(1) [20]. This is well reflected in water adsorption experiments carried out on DUT-98(3) in which the uptake is found to decrease upon heating of the powder at 80 °C in dynamic vacuum (Figure 5c). Although no adsorption-induced transitions could be observed in downsized DUT-98 crystals, the samples showed a high sensitivity towards elevated temperature in vacuum – conditions found to initiate an irreversible structural contraction. This finding implicates that guest-free DUT-98*op* is a metastable phase and DUT-98*cp* or *ht* phase is the thermodynamic stable phase observed upon solvent removal in DUT-98(1) and partially in DUT-98(2)–(4). Thus, smaller crystal sizes seem to stabilize the presence of a metastable *op* phase by impacting the activation barrier, presumably due to the contribution of the surface energy and other factors. This observation was previously made for DUT-8 for which the guest-free metastable *op* phase was also found to be stabilized upon crystal downsizing. The reports on shape–memory effects [19,42] in flexible MOFs found to be intrinsically connected to the formation of metastable states indicate that crystal size and morphology are in fact parameters that significantly alter the free energy landscape of bistable adsorbents and therefore also impact the adsorption behavior.

## Conclusion

In conclusion we demonstrated that the adsorption behavior and structural transition of the flexible Zr-MOF DUT-98 strongly depends on the size of the crystals. In addition, we demonstrate that cycled adsorption experiments can have a large impact on the properties of flexible MOFs by altering the crystal morphology, size and mosaicity. In many regards, DUT-98 is found to behave similar to DUT-8(Ni) and the applied methods indicate changes in the activation energy upon crystal downsizing to be responsible for the observed behavior. The lack of adsorption-induced structural transition for nanometer-sized crystals was previously described also for ZIF-8 [13] and DUT-49 [9]. However, the mechanism and structural transition in these flexible MOFs are of a different nature and therefore cannot be directly compared with DUT-98. After all, there might not be a single theory for explaining the effects of crystal size variation on the adsorption behavior of flexible MOFs. Only further crystal-size-dependence analysis of novel flexible MOFs with different transition and structures can solidify the presented observations and help to postulate a detailed mechanism. Furthermore, the effect of microscopic defects upon changes in the synthesis procedure should not be neglected as an increasing number of defects has been demonstrated to strongly alter the mechanical stability of MOFs [43–45]. In accordance, novel experimental investigations on flexible MOFs should consider crystal size and cycling effects and include these in experiments and discussion. In addition, we would like to motivate computational chemists to extend recent efforts [46] in developing strategies to analyze these phenomena in silico.

## Supporting Information

File 1 Synthetic procedures and additional characterization of the discussed compounds.
