# Supplementary material for: The impact of crystal size and temperature on the adsorption-induced flexibility of the Zr-based metal–organic framework DUT-98
Source: Beilstein J Nanotechnol. 2019 Aug 20;10:1737–44. doi: 10.3762/bjnano.10.169 (PMC6719731; doi:10.3762/bjnano.10.169)
Supplement: File 1 — Synthetic procedures and additional characterization of the discussed compounds. [file Beilstein_J_Nanotechnol-10-1737-s001.pdf]

BEILSTEIN JOURNAL OF NANOTECHNOLOGY

Supporting Information

for

The impact of crystal size and temperature on the adsorption-induced flexibility of the Zr-based metal–organic framework DUT-98

Simon Krause, Volodymyr Bon, Hongchu Du, Rafal E. Dunin-Borkowski, Ulrich Stoeck, Irena Senkovska and Stefan Kaskel

Beilstein J. Nanotechnol. **2019,** *10,* 1737–1744. doi:10.3762/bjnano.10.169

Synthetic procedures and additional characterization of the discussed compounds

License and Terms: This is a supporting information file under the terms of the Creative Commons Attribution License (http://creativecommons.org/licenses/by/4.0). Please note that the reuse, redistribution and reproduction in particular requires that the authors and source are credited.
The license is subject to the *Beilstein Journal of Nanotechnology* terms and conditions: (https://www.beilstein-journals.org/bjnano)

1. Materials and Methods

The following chemicals and methods were applied in the synthesis and analysis of DUT-98 [S1]:

$ZrCl_4$ (Sigma Aldrich), *N,N*-dimethylformamide anhydrous (<150 ppm H_2O, Sigma Aldrich), acetic acid (Fisher Scientific) and ethanol (abs.) (VWR Prolabo) were used as received. The Ligand H3CPCDC was used from a previous study [S1,S2].

Powder X-ray diffraction (PXRD) patterns were collected with a PANALYTICAL X'Pert Pro diffractometer operated at 40 kV and 30 mA with monochromatic Cu $K\alpha_1$ (λ = 0.15405 nm) radiation. The samples were placed on a Si sample holder. Variable temperature PXRD was performed in a closed chamber under dynamic vacuum of 10^{-4} mbar on a PANALYTICAL X'PERT PRO with λ = 0.15405 nm in Bragg–Brentano-geometry. The temperature was increased with 5 K min^{-1} steps starting at 300 K. For each selected temperature, PXRD patterns in the 2Θ-range from 4 to 22°, with a step size of 0.028° and 40 s exposure per step were recorded. Gas and vapor adsorption experiments were carried out on a Belsorp-max instrument (MicrotracBEL Corp., Japan). High purity gases were used (N_2: 99.999%, He: 99.999%). Water adsorption isotherms were recorded at 298 K using a Hydrosorb instrument from Quantachrome Instruments. Thermogravimetric analysis (TGA) was carried out in air atmosphere using a Netzsch STA 409 thermal analyzer. Scanning electron microscopy (SEM) and Scanning electron transmission microscopy (STEM) images were taken with secondary electrons in a HITACHI SU8020 microscope. Diffuse reflectance infrared Fourier transform (DRIFT) spectra were recorded on a Bruker Vertex 70 spectrometer with 16 scans for each sample and a resolution of 2 cm^{-1}. Prior to the measurement 3–5 mg of sample were mixed with 60 mg of dry KBr and pressed in the sample holder. TEM images were recorded with a binning of 4 at 80 kV accelerating voltage using an FEI Titan G3 50-300 PICO microscope equipped with a 4096 x 4096 pixels Gatan Ultrascan 4000 UHS charge coupled device camera (CCD). The frame exposure time is 0.3 s.

2. Synthesis and activation procedure

General synthesis of DUT-98

Synthesis and activation of DUT-98

Synthesis of DUT-98 samples with varying crystal size was performed as following:

Synthesis of DUT-98(1)

The synthesis was performed according to a previously reported procedure [S1].

Synthesis of DUT-98(2)

In a 100 mL Schott bottle $ZrCl_4$ (248 mg, 1.06 mmol) and acetic acid (16 mL, 270 mmol) are dissolved in 30 mL of a 1:1 mixture of DMF and *N*-methyl-2-pyrrolidon at 80 °C for 3 h. To the solution the ligand H_3CPCDC (200 mg, 0.53 mmol) is added and dissolved by ultrasonication. The reaction mixture is kept at 120 °C for 2 d to form a white precipitate.

Synthesis of DUT-98(3)

In a 100 mL Schott bottle $ZrCl_4$ (145 mg, 0.6 mmol) and acetic acid (4 mL, 70 mmol) are dissolved in 18 mL of a 1:1 mixture of DMF and *N*-methyl-2-pyrrolidon at 80 °C for 3 h. To the solution the ligand H_3CPCDC (120 mg, 0.3 mmol) is added and dissolved by ultrasonication. The reaction mixture is kept at 120 °C for 18 h to form a white gel.

Synthesis of DUT-98(4)

In a 100 mL Schott bottle $ZrCl_4$ (370 mg, 1.6 mmol) and acetic acid (10 mL, 175 mmol) are dissolved in 45 mL of DMF. To the solution the ligand H_3CPCDC (300 mg, 0.8 mmol) and water (5 mL, 280 mmol) are added and dissolved by ultrasonication. The reaction mixture is kept at 120 °C for 16 h to form a white gel.

Synthesis of DUT-98(Hf)

In a 20 mL Pyrex tube 256 mg (0.8 mmol) $HfCl_4$ and acetic acid (0.5 mL, 8.75 mmol) are dissolved in 5 mL of DMF. To the solution the ligand H_3CPCDC (60 mg, 0.16 mmol) and water (0.2 mL, 11.2 mmol) are added and dissolved by ultrasonication. The reaction mixture is kept at 120 °C for 24 h to form a white gel.

An overview of the synthesis parameter is provided in the following table.

Table S1: Reaction conditions.

Material ID	solvent	eq. water(a)	eq. acetic acid(a)	Reaction time	Yield (%) (a)	Crystal size(b)
**DUT-98(1)** [S1]	DMF	<0.01	1625	14 d	46	120 µm
**DUT-98(2)**	DMF:NMP (1:1)	<0.02	509	2 d	49	10 µm
**DUT-98(3)**	DMF:NMP (1:1)	<0.02	233	18 h	52	500 nm
**DUT-98(4)**	DMF	350	219	16 h	51	50 nm
**DUT-98(Hf)**	DMF:NMP (1:1)	70	55	24 h	55	130 nm

(a) Based on linker H_3CPCDC, (b) Estimated from SEM images, size refers to length of the rod-shaped crystals due to crystal intergrowth no distribution could be determined because the error is estimated to be 200%.

After solvothermal synthesis the powders were washed with fresh DMF 4 times over a period of 2 days, and suspended in ethanol which was exchanged 6 times for 4 days. A Jumbo Critical Point Dryer 13200J AB (SPI Supplies) was used for supercritical drying. The sample was purged 20 times with liquid CO_2 (purity: 99.995%) at ~15 °C over 96 hours before raising the temperature and pressure beyond the critical point and releasing CO_2.

Table S2: BET surface area, specific pore volume and uptake of nitrogen of the series of investigated DUT-98 samples.

	**BET Surface area (m^2 g^{-1})**	**N_2-uptake at p/p_0 = 0.1 (cm^3 g^{-1})**	**N_2-uptake at p/p_0 = 0.98 (cm^3 g^{-1})**	**Specific pore volume at p/p_0 = 0.98 (cm^3 g^{-1})**	**Specific micropore volume at p/p_0 = 0.1 (cm^3 g^{-1})**
**DUT-98(1)**	21	21	257	0.4	0.005
**DUT-98(2)**	954	232	258	0.41	0.37
**DUT-98(3)**	1303	324	442	0.95	0.53
**DUT-98(4)**	1161	289	1088	1.66	0.49
**Simulated[(a)]**	1665	464	464	n. a.	0.72
**DUT-98(Hf)**	1004	209	674	1.31	0.41

(a) calculated on the basis of DUT-98(Zr)*op* crystal structure and using the software POREBLAZER.

A change in crystal morphology was observed upon performing multiple adsorption-desorption experiments on the same DUT-98(1) sample. Isotherms of these experiments can be obtained from reference [S1] and were performed in the following order: nitrogen 77 K, CO_2 195 K, *n*-butane 273 K, and vapors of methanol, ethanol, 2-propanol, toluene, heptane, acetone, and water all at 298 K.

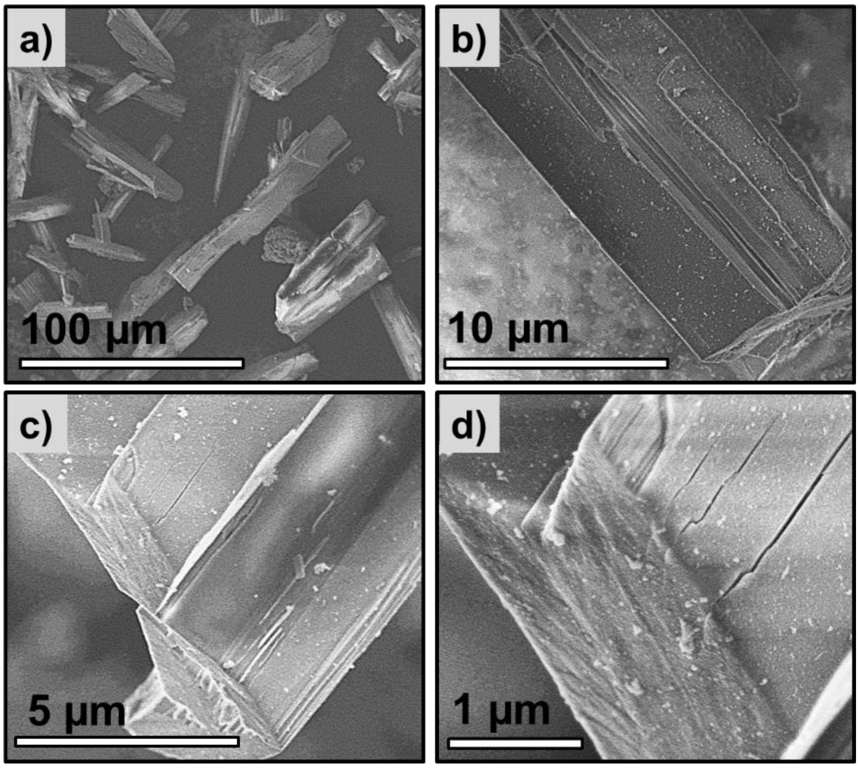

Figure S1: SEM images of DUT-98(1) at different magnifications after supercritical activation before adsorption cycling.

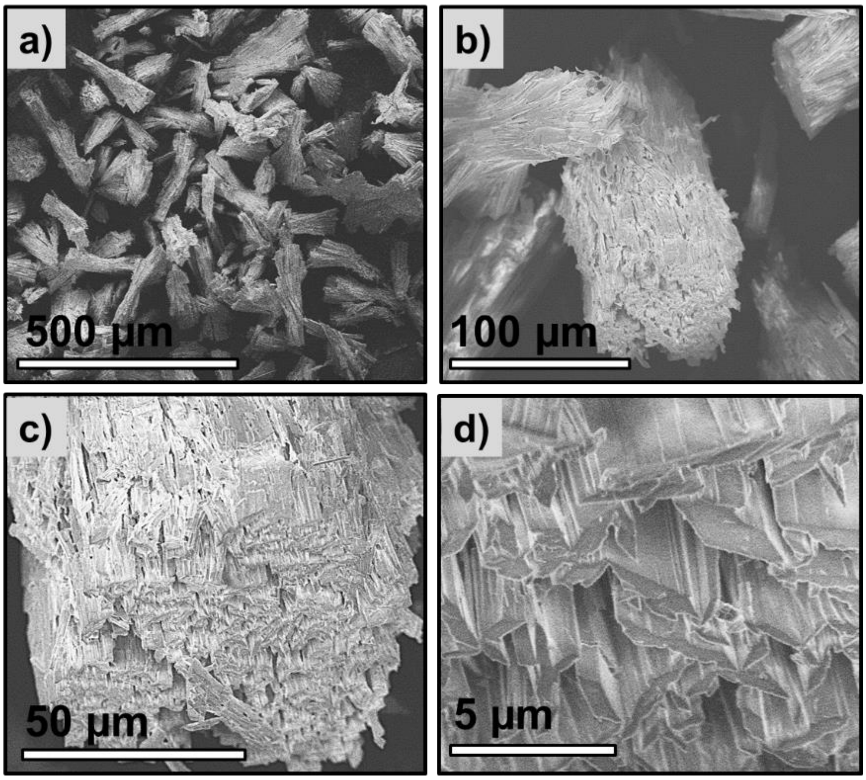

Figure S2: SEM images of DUT-98(1) at different magnifications after adsorption cycling.

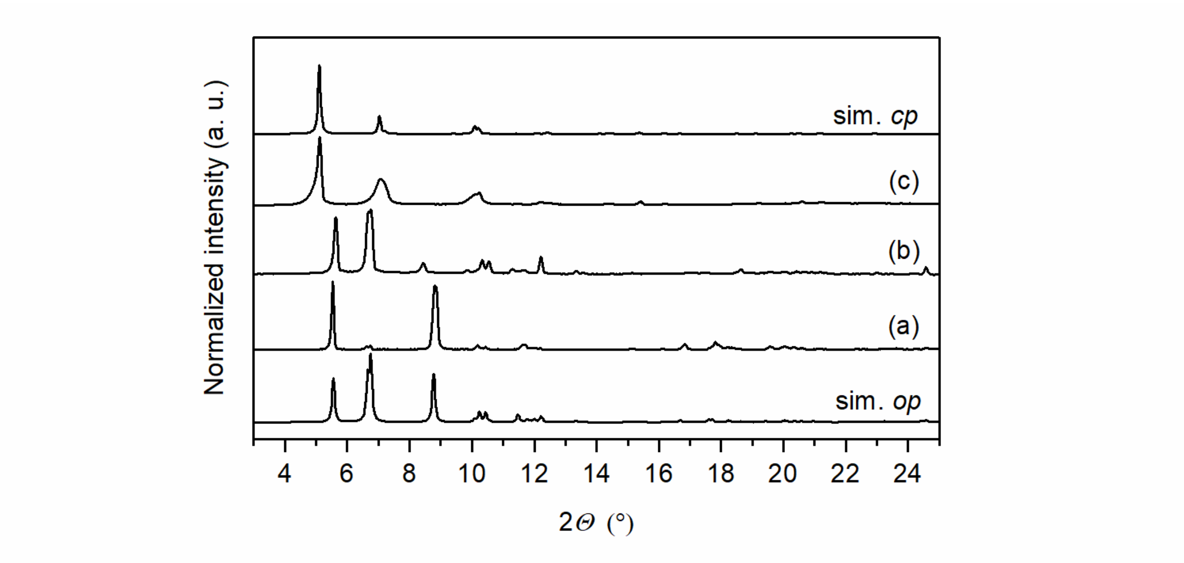

Figure S3: PXRD patterns of a) DUT-98 after supercritical activation, b) after adsorption cycling, c) suspended in methanol at 298 K with sample placed in sealed glass capillary. Simulated patterns for DUT-98*op* and *cp* are given for comparison.

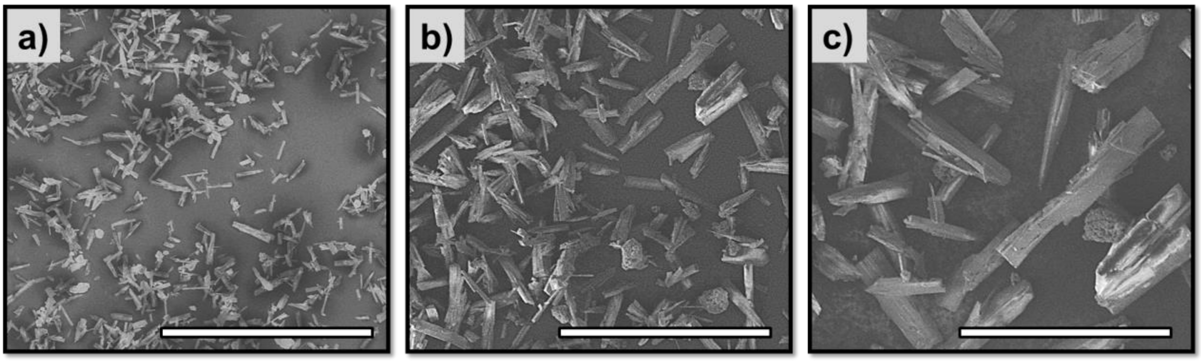

Figure S4: SEM images of supercritically activated DUT-98(1). Scale bars a) 500 µm, b) 200 µm, c) 100 µm.

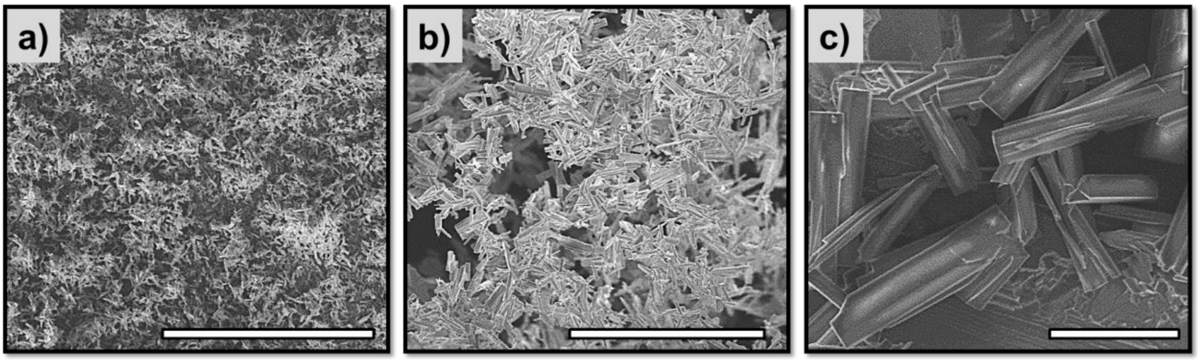

Figure S5: SEM images of supercritically activated DUT-98(2). Scale bars a) 200 µm, b) 50 µm, c) 5 µm.

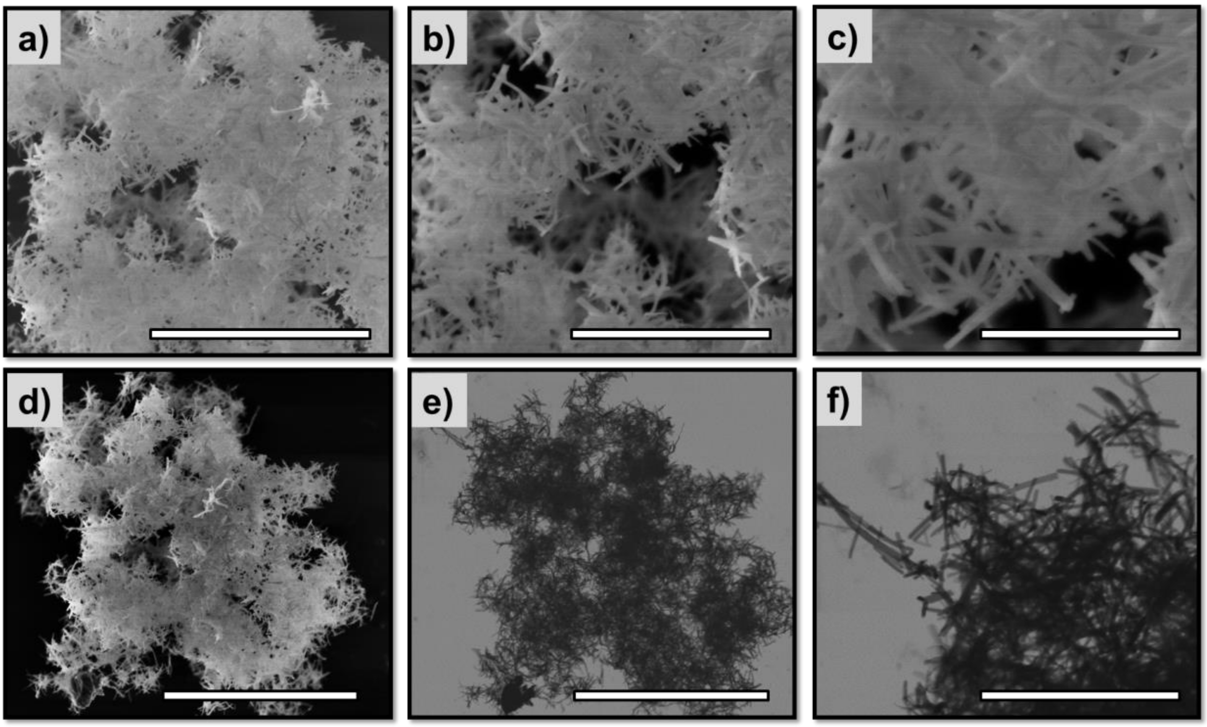

Figure S6: a–d) SEM images of supercritically activated DUT-98(3). e,f) STEM images of supercritically activated DUT-98(3). Scale bars a) 3 µm, b) 1 µm, c) 500 nm, d) 3 µm, e) 3 µm und f) 1 µm.

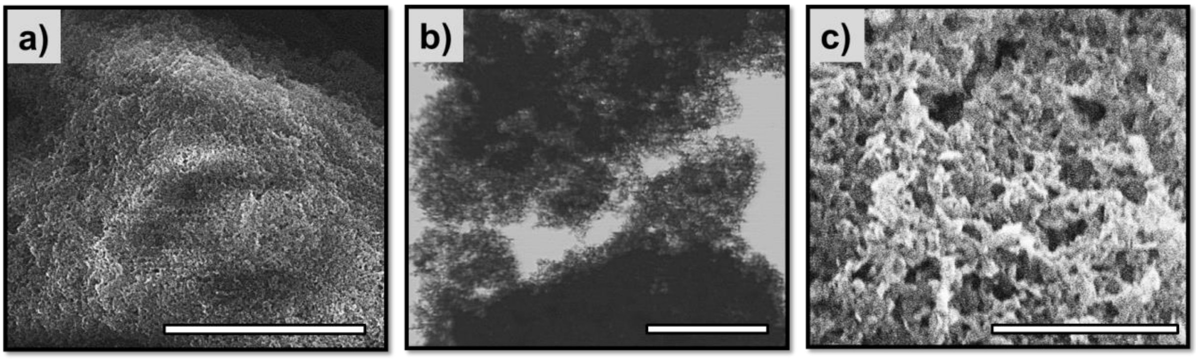

Figure S7: SEM images of supercritically activated DUT-98(4). Scale bars a) 3 µm, b) 1 µm, c) 500 nm.

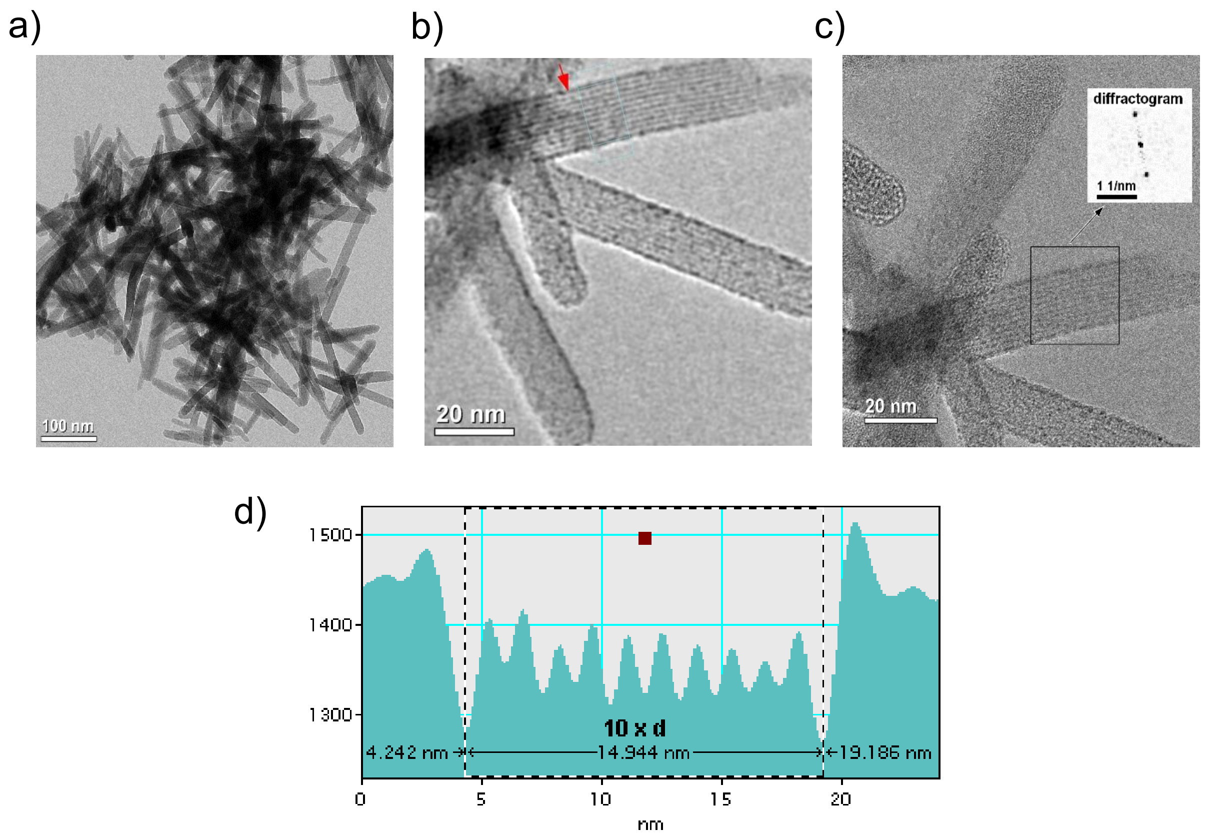

Figure S8: TEM images of DUT-98(Hf). Scale bars a) 100 nm, b) 20 nm, c) 20 nm. These images were recorded with focus and spherical aberrations both negative, therefore the lattice planes comprising the Hf clusters show dark contrast in the images. Image shown in b) was filtered using a nonlinear algorithm [S3] for noise reduction. The averaged lattice space observed in HRTEM images shown in b) and c) is about 1.5 nm as revealed in d) by the line profile across the nanocrystal indicated by the red arrow in b).

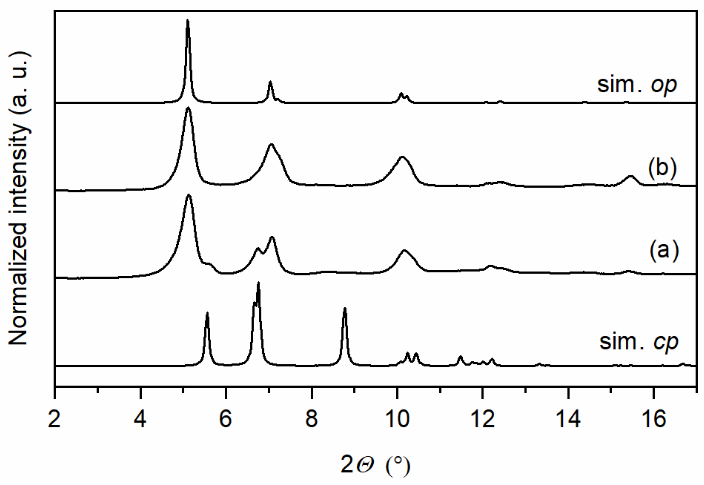

Figure S9: PXRD patterns of DUT-98(Hf) a) after supercritical activation, and b) suspended in ethanol. Simulated patterns derived from single crystal structure for DUT-98(Zr)*op* and *cp* are given for comparison.

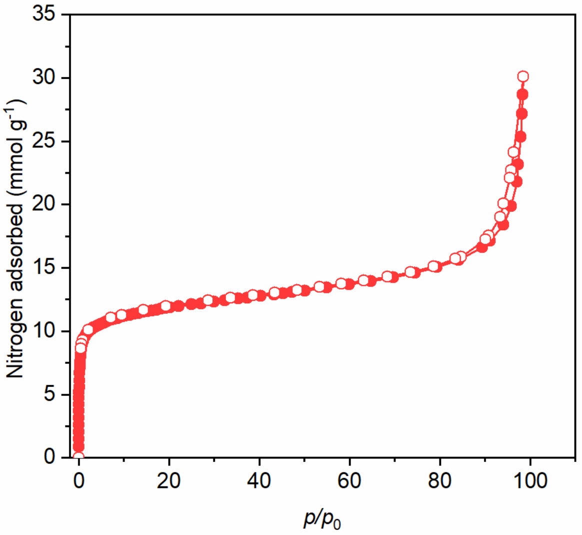

Figure S10: Nitrogen adsorption isotherm at 77 K of DUT-98(Hf). Closed symbols adsorption, open symbols desorption.

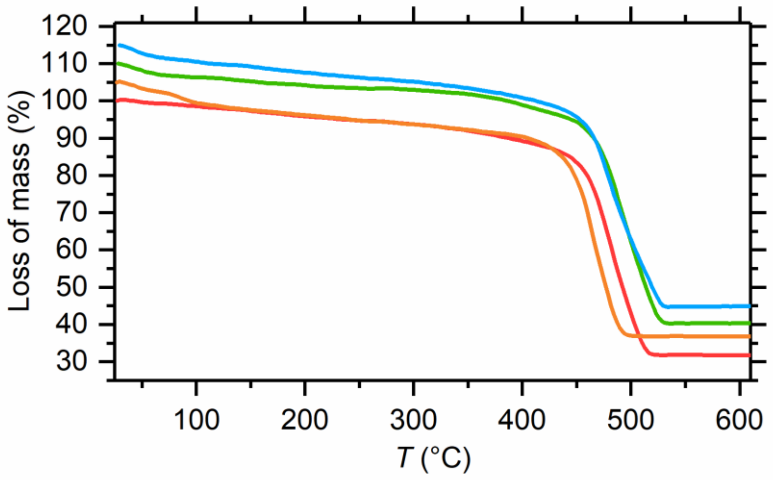

Figure S11: TGA analysis of DUT-98(1) (red), DUt-98(2) (organe), DUT-98(3) (green), DUT-98(4) (red) with 5 °C offset.

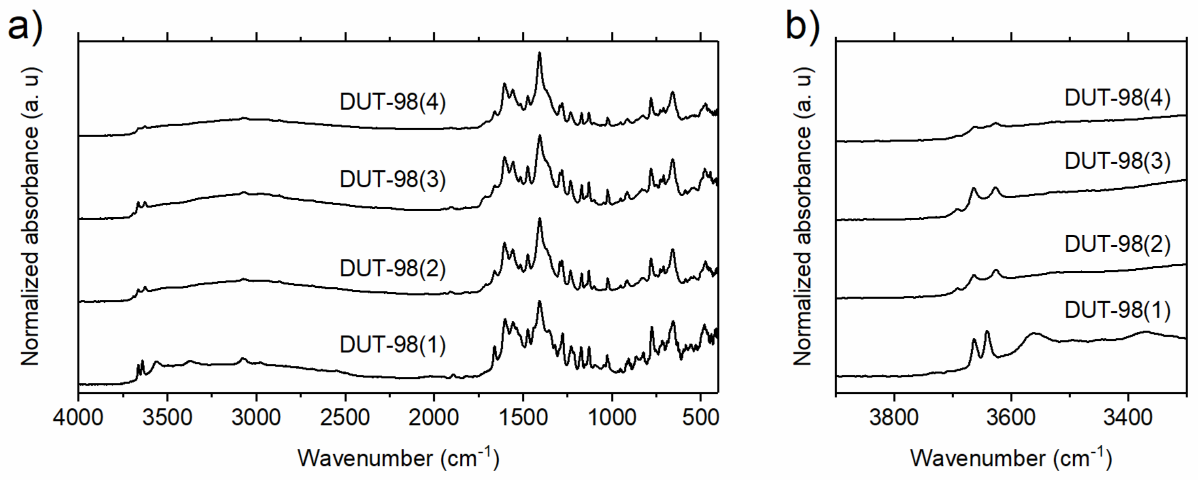

Figure S12: a) DRIFT spectra of DUT-98 and b) Magnified section of water-relevant wavenumbers.

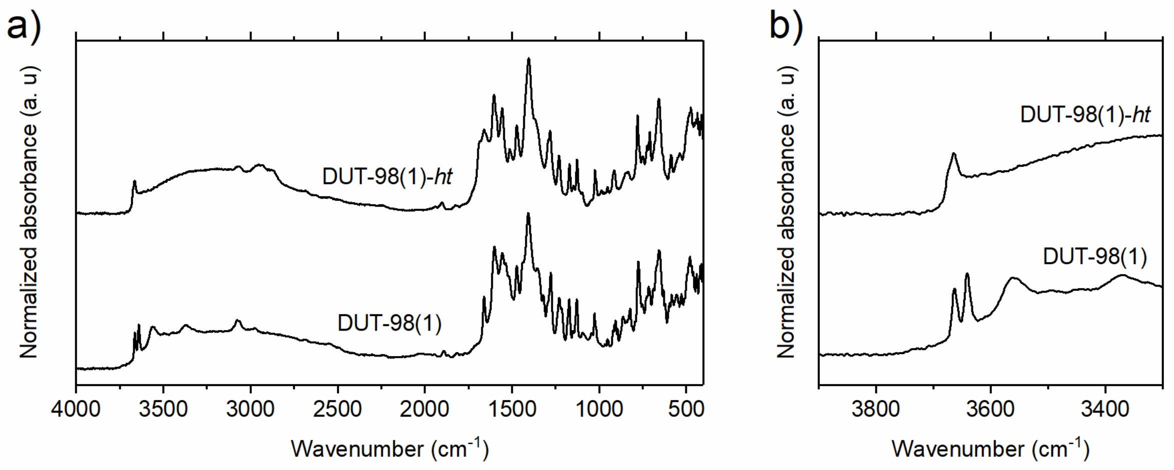

Figure S13: a) DRIFT spectra of DUT-98(1) and DUT-98(1)-*ht* after treatment at 80 °C in vacuum. b) Magnified section of water-relevant wavenumbers.

References

S1 Krause, S.; Bon, V.; Stoeck, U.; Senkovska, I.; Többens, D. M.; Wallacher, D.; Kaskel, S. *Angew. Chem. Int. Ed.,* **2017**, *56*, 10676-10680. doi:10.1002/anie.201702357

S2 Stoeck, U.; Senkovska, I.; Bon, V.; Krause, S.; Kaskel, S. *Chem. Commun.,* **2015**, *51*, 1046-1049. doi:10.1039/C4CC07920E

S3 Du, H. *Ultramicroscopy,* **2015**, *151*, 62-67. doi:10.1016/j.ultramic.2014.11.012
